# Development of a Mixed Hypnosis and Music Intervention Program for the Management of Pain, Anxiety, and Wellbeing in End-of-Life Palliative Care

**DOI:** 10.3389/fpain.2022.926584

**Published:** 2022-07-06

**Authors:** Josiane Bissonnette, Stephica Pierre, Anh Thu Julia Duong, Anne-Marie Pinard, Pierre Rainville, David Ogez

**Affiliations:** ^1^Département d'anesthésiologie et de Médecine de la douleur, Université de Montréal, Montréal, QC, Canada; ^2^Faculté de Musique, Université Laval, Laval, QC, Canada; ^3^Département de psychologie, Université du Québec à Montréal, Montréal, QC, Canada; ^4^Department of Medicine, McGill University, Montréal, QC, Canada; ^5^Département d'anesthésiologie et de soins intensifs, Université Laval, Laval, QC, Canada; ^6^Centre intégré de recherche en réadaptation et intégration sociale (CIRRIS), CIUSSS de la Capitale-Nationale, Quebéc, QC, Canada; ^7^Département de stomatologie, Université de Montréal, Montréal, QC, Canada; ^8^Centre de Recherche, Institut universitaire de gériatrie de Montréal (CRIUGM), Montréal, QC, Canada; ^9^Centre de Recherche, Hôpital Maisonneuve-Rosemont (CR-HMR), Montréal, QC, Canada

**Keywords:** pain, anxiety, hypnosis, music, complementary intervention, intervention development, wellbeing, palliative care

## Abstract

**Background:**

The palliative care people present needs that can be partially met by complementary intervention. Approaches based on the use of hypnosis and music are increasingly being studied and have shown potential benefits on pain, anxiety, and wellbeing for many populations including those in palliative care.

**Objective:**

This study aims to present the initial process of creating and refining a hypnosis and music intervention program intended for persons in palliative care, with a panel of experts of diverse relevant backgrounds. It also aims to evaluate its feasibility, preliminary acceptability, and content.

**Methods:**

To achieve the objectives, we followed ORBIT recommendations for the development and redesign of behavioral interventions (phase I a-b). Based on a meta-analysis, reference interventions were identified and then adapted to the target population. Twenty-two experts from different backgrounds were consulted to obtain their evaluation on the acceptability, feasibility, and content of the interventions.

**Result:**

The various components of the program were deemed appropriate or very appropriate by over 80% of the experts. However, possible risks were raised related to some uncertainty about the reactions of individuals to the intervention. Several experts (32%) indicated potential adverse effects consisting of negative emotional experiences during the sessions. Modifications were proposed specifically to reduce or mitigate this risk. Over 90% of the experts considered that the revised program provides a safer and more appropriate intervention for palliative care persons.

**Conclusion:**

A mixed intervention program with hypnosis and music has been developed and attained a high level of consensus by the experts. The proposed intervention is ready to be assessed for clinical efficacy in a pilot study (ORBIT Phase II).

## Introduction

The quality of life of patients in end-of-life palliative care is a fundamental issue for them and their families. It is at the heart of the comfort care provided at this stage of life (1). Fifty to seventy percent of cancer patients in England wish to die at home (2). Their choice to live these last moments in their residence meets an important need. However, this approach is demanding and refers to issues that people in palliative home care face on a daily basis. Palliative care mobile teams may be confronted with patient anxiety, pain and suffering as well as the presence of medication-related side effects (1, 3, 4). Other organizational challenges refer to difficulties in accessing resources and services at all times of the day (5).

To address the challenges related to the quality of end-of-life care, there is a variety of complementary non-pharmacological interventions (6). A complementary approach refers to “a non-mainstream approach used together with conventional medicine” (7). Among these proposals, interventions based on music and hypnosis are relevant.

Music intervention is an umbrella term referring to an intervention using music. Music medicine intervention is defined as “listening to pre-recorded music, offered by medical staff” (8). Music medicine differs from music therapy in that it does not focus on the therapeutic relationship between a provider and a patient (9). Several disciplines, including music therapy, community music, music education, daily use of music, and music medicine, may involve interrelationships between music, health, and wellbeing (10). The musical intervention developed here is part of the conceptual framework of music medicine and belongs to the category of complementary approaches, contrary to music therapy which is considered as a discipline. The musical approach we chose is based on previous studies demonstrating the superiority of self-selected or preferred music over standard selected music in improving pain and anxiety outcomes in a variety of settings (11–14). It also takes into consideration musical features to better accompany pre-recorded text (15).

Hypnotherapy is defined by The American Psychological Association Executive Committee, Division 30, as “the use of hypnosis in the treatment of a medical or psychological disorder or concern“ and hypnosis as “a state of consciousness involving focused attention and reduced peripheral awareness characterized by an enhanced capacity for response to suggestion” (16). Music and hypnosis present little risk of side effects and can be self-administered at different times of the day and at different stages of the disease. They enhance the quality of life of different populations, acting on pain, anxiety, and wellbeing (8, 17–24).

A meta-analysis on hypnosis and music in a palliative care context observed a significant decrease in pain with an effect size of *d* = −0.42, *p* = 0.003 for randomized controlled trial studies (*k* = 4) (25). Analyses of the results of pre-post changes in hypnosis (*k* = 3), preferred music (*k* = 3), and music/hypnosis (*k* = 2) report an improvement in pain, anxiety, and wellbeing. Their acceptability and feasibility achieved a high level of satisfaction. These positive results should be interpreted with caution given the limited number of available studies testing music and hypnosis interventions developed with a rigorous protocol for people in palliative care. This justifies further development and testing of such interventions in various settings.

The Obesity-Related Behavioral Intervention Trials (ORBIT) model proposes four phases to develop a rigorous behavioral intervention program: design (phase Ia: define; phase Ib: refine); preliminary tests (phase II); test effectiveness (phase III); overall effectiveness (phase IV) (26).

The purpose of this study was to complete the design of a mixed hypnosis and music intervention program (ORBIT Phases Ia and Ib). Specifically, it aimed to assess the feasibility, preliminary acceptability, and content of the program for home-based palliative care patients, and to gather expert recommendations for improving the program for pilot testing.

## Methods

### Development and Design of the First Version of the Mixed Intervention Program in Hypnosis and Music (ORBIT-Phase Ia)

The first stage of this project was to carry out the ORBIT-Phase Ia. It consists in defining the intervention program from the data of the literature and in developing the first version of a program.

#### Intervention Milestones

To establish the milestones of the intervention, we conducted a meta-analysis (25), we consulted papers on the effect of specific musical components and clinical reference literature on hypnosis.

We analyzed the strengths and weaknesses of selected interventions and identified flagship ones by assessing their feasibility and effect size. We retained three studies (20, 21, 23). Coelho and Gutgsell's interventions included, respectively, one and two intervention sessions; they had good feasibility and fidelity, and effect sizes for pain reduction are large (*d* = −1.58 and *d* = −0.76). Each session ranged in length from 13 to 20 min, with a high recruitment and low attrition rates. Both contain musical and hypnotic components. Coelho's intervention was accompanied by “relaxing music” and included an introduction, exercises of breathing and muscle relaxation, images of a comfortable place, and suggestions for wellbeing. This intervention was built according to the recommendations of the UK Medical Research Council (27). Gutgsell's intervention included preparation for the intervention, autogenic relaxation (breathing, relaxation), music at the same time as the patient explored a safe place, and a conclusion. The musical part contained various slow soft pieces and included improvisation in G myxolydian. Peng's music intervention consisted, first, of identifying the patient's musical preferences and, second, of playing the selected preferences. The acceptability and effect sizes of this intervention on pain (*d* = −3.81, *p* < 0.001) and anxiety (*d* = −3.31, *p* < 0.001) were very large (25).

To identify preferred musical characteristics in background music, we found a study that evaluated different musical components of musical frames accompanying pre-recorded spoken text. The results reported that musical accompaniments with a high degree of harmonic and melodic simplicity enabled a greater state of mindful state to be achieved during guided meditation with music (15).

To determine the content of the suggestions and metaphors, we consulted a clinical manual, frequently cited in scientific articles (28), as well as a script written and used in clinical practice by one of the authors (HYlaDO^©^, DO, psychologist and hypnotherapist)^1^.

#### Intervention Development and Description

Based on the results of a meta-analysis (25), based on a clinical script (28)^1^, and on studies on musical components (11–15), we developed the first version of a mixed intervention program that integrates hypnosis and music. In keeping with the principles of pragmatic studies and to foster respect for the individuality of patients according to the principles of person-centered care (29, 30), we offer three choices of interventions, which they can select according to their preferences: Hypnosis (H), Music (M), and Hypnosis with Music (HM). Inspired by Coelho et al.'s (20) and Gutgsell et al.'s (21) interventions, we determined that each intervention would consist of two sessions ranging from 15 to 30 min in length.

During the intervention setup, a health care provider is responsible for preparing the patient to receive the intervention in the best environmental and physical conditions possible. He/she may suggest taking a comfortable position, dim the lights, close or open the curtains, create a pleasant atmosphere, and try to reduce surrounding noise as much as possible. Then, he/she ensures that the patient is comfortable listening to the pre-recorded session.

All three interventions follow the same structure, with some variations (see Table 1). Intervention setup, introduction, induction, deepening, and emergence are similar for all the interventions. The main distinction between them (H, M and HM) lies in the transformation section. Instead of listening to metaphors, participants who chose an intervention with music (M or HM), listen to a piece of music that they enjoy. Background music also accompanies the music interventions.

**Table 1:** Structure of the MuzHyp^©^ intervention program (version 1.0).

	**Interventions**				
	**Hypnosis (2 sessions)**	**Music (2 sessions)**		**Hypnosis and music (2 sessions)**	
Before the intervention (Health caregiver or beneficiary attendant)	Setting up of the intervention	Setting up of the intervention		Setting up of the intervention	
**Pre-recorded sessions**					
Introduction	Welcome and presentation of the intervention	Welcome and presentation of the intervention		Welcome and presentation of the intervention	
Induction	Respiration Relaxation	Respiration Relaxation	Soft background music in myxolydian	Respiration Relaxation	Soft background in myxolydian
Deepening	Counting	Counting		Counting	
Transformation	Pleasant place Metaphor 1 (horse) (session 1) Metaphor 2 (island) (session 1) Reflection (session 2) Positive hand exercise (session 2)	Music chosen by the patient		Pleasant place Music chosen by the patient	
Post-hypnotic suggestions	Post-hypnotic suggestions	N/A	Soft background in myxolydian	Post-hypnotic suggestions	Soft background in myxolydian
Emergence	Emergence with countdown	Emergence with countdown		Emergence with countdown	

The introduction presents the sequence of the session. The induction first involves the direction of the patient's attention on a fixed point, then for a moment, directs the attention toward respiration, “As you focus, you begin to direct your attention to your breathing, inhale and exhale at your own pace…”. This is followed by suggestions of relaxation of the body “(…) as if the relaxation, in fact, goes down from the face to the lower body, a bit like a wave of relaxation (…) and maybe you can feel the heaviness of the body as well (…).” This section continues toward a 10-0 count where the patient is invited to deepen his/her state of wellbeing, and physical and psychological relaxation. In the interventions containing hypnosis (H, HM), patients are invited to experience and live a moment of wellbeing in a pleasant place.

The hypnosis intervention without music then narrates metaphors that are not present in the other two interventions. The “horse metaphor” tells the story of a child who rides a horse to his grandfather's house. One day, he gets completely lost and leaves the reins of his horse to return home. In this metaphor, it is suggested to trust one's body as the child trusts the horse. The metaphor of the island tells of a person's journey through different places to get to an island. He can choose a fast, but more laborious path or a slower path, and take his time. For its part, the reflection section of the second hypnosis session is designed to describe a moment when the individual looks at a reflection in the water and then realizes that it is their own. Finally, the “positive hand metaphor” consists of putting comfort, beautiful moments of his life, wellbeing, and a balm in a hand chosen by the patient. This positive hand is then placed on a less comfortable area of the patient and the feeling of wellbeing is transferred to the uncomfortable area and to the whole body.

The metaphors/techniques (safe place, positive hand, pain modulation) were selected from textbooks on clinical hypnosis according to the purpose of this program (to decrease pain, anxiety and improve wellbeing) (28). Other metaphors (horse and reflection) were defined by the hypnotherapist authors (JB, DO) for this project, taking into account the recommendations of hypnosis manuals (28)^1^.

The two interventions with music (M and HM) involved a selection of preferred music by the patient and was inspired by Peng's study (23). The interventions with music also incorporate a soft musical background. Taking into consideration the results of Dvorak's study and some musical elements of Coelho's and Gutgsell's interventions, we created a musical framework in myxolydian with a simple melodic structure, taking care not to overload the listener (15, 20, 21). The tempo was undefined and very slow (fewer than 60 beats per minute), and the harmonic changes were relatively infrequent.

The recording of the text and music of each session was done by the first author (JB) and served as a prototype for the evaluation process. An intervention manual was created and includes the procedure to follow by health care providers for implementing the intervention. It also included the verbatim of each intervention session.

### Evaluation Process of the Intervention Program (ORBIT Phase 1b)/Objective

Phase 1b involves evaluating the first version of the intervention described above and making the appropriate modifications to ensure acceptability, feasibility, and content validity from a diversity of perspectives.

#### Evaluators/Experts

We invited a group of experts from Quebec, also identified here as “evaluators,” to critically review the first version of the intervention program. Experts are defined as “someone who possessed the relevant knowledge and experience and whose opinions are respected by fellow workers in the field” (31). To ensure a portrait of all the parties involved, we opted for a stratified voluntary sampling panel (32). The team of evaluators was composed of 22 individuals (questionnaire 1) and 20 individuals (questionnaire 2). It was composed of health service managers, hypnotherapists, nurses, physicians, music therapists, beneficiary attendant, family caregivers, social workers, speech therapist and psychologists. In the results section, participants' verbatim are identified by a code corresponding to them (see Table 2). They came from five different regions in the province of Quebec, Canada.

**Table 2:** Profile of evaluators.

**Categories**	**Description of expertise in palliative care**	***n* (%)**
Gender (*n* = 22)		
Male		4 (18%)
Female		18 (82%)
Age (*n* = 22)		
18–34		3 (14%)
35–49		13 (59%)
50–64		2 (9%)
65 et +		4 (18%)
Region (*n* = 22)		
Québec		6 (27%)
Chaudière-Appalaches		10 (45%)
Laval		1 (5%)
Laurentides		1 (5%)
Mauricie		1 (5%)
Montréal		3 (14%)
Professions/competency^a^		
Health service manager (C1-C3)	Including coordination of at-home services	3
Hypnotherapist (HT1-HT6)	General practice^b^	6
Nurse (N1-N4)	N1-N4 Palliative care practice	5
Physicians (P1-P3)	Palliative care practice (P1-P2); Psychiatrist (P3)	3
Music therapist (MT1-MT2)	Palliative care practice (MT1). General practice (MT2)	2
Beneficiary attendant (BA)	General practice	1
Family caregiver (FC1-FC4)	Family caregiver for a person who has been or is currently in palliative care	4
Social worker (SW1-SW2)	Palliative care practice	2
Speech therapist (ST)	Other specialty	1
Psychologist (Psy1-Psy2)	General practice	2

a *Each evaluator may have more than one profession or skill, the percentages are therefore not calculated for this category*.

b *The term “general practice” is used to refer to practice that is not directed exclusively toward palliative care*.

Two experts did not respond to the invitation to answer the second questionnaire. One expert mentioned that he had a busy schedule and did not have time to complete it. The evaluators included various age groups, with a majority (59%) between the ages of 34–49.

#### Ethics Committee Approval

The research project was approved by the Ethics Committee of the University of Montreal (# 2021-1243) and CISSS-Chaudière-Appalaches (CISSS-CA; # 2022-896). Each expert evaluator signed a consent form to participate in the project. No monetary compensation was provided.

#### Procedure

A validation process of the intervention protocol was conducted with the evaluators using the principles underlying the Delphi method.

Delphi method is a classical gold-standard approach for systematically collecting and aggregating the judgment of a group of experts on specific issues and problems to obtain a consensus of opinions (33, 34). It uses a series of questionnaires, each containing a summary of the responses and feedback from previous questionnaires. Delphi thereby offers experts the opportunity to modify or refine their responses at each stage. This method has the advantage of preserving the anonymity of the evaluators from each other, reducing potential biases associated with social acceptability, group dynamics and hierarchical status (social, professional or organizational), while allowing every evaluator to react to others' comments in the second iteration of the consultation. Finally, it allows individuals from different backgrounds to participate in the process, regardless of their respective availability.

The objective of the expert consultation was to validate and obtain consensus on (1) the feasibility and acceptability of the program and (2) the content of the intervention program.

#### Data Collection

The evaluators were asked to read the protocol and to listen to the recordings of the interventions. Considering the scope of the intervention program and to keep as many evaluators as possible by not overloading them, evaluators could choose one or more intervention(s) to evaluate between the following: hypnosis intervention (*n* = 19), music intervention (*n* = 13), and hypnosis and music intervention (*n* = 14). Those who wished to listen to the interventions with music could select the piece of their choice from a list or request a version containing their specific musical preference. Two rounds of questions were conducted to reach a consensus before making any changes to the interventions. The questionnaires were constructed by the first three authors (JB, SP, AD). After obtaining the responses to the first questionnaire, the results were summarized and presented to the experts in the second questionnaire. They were then asked to give their opinion on new suggestions and to re-evaluate their position on some of the non-consensual items from the first questionnaire. The steps in the evaluation process are summarized in Table 3.

**Table 3:** Steps for conducting the evaluation process.

1. Send out emails through associations, social networks, health service organization, and professional contacts to recruit potential evaluators. • Interested evaluators are invited to respond to the announcement and leave their contact information.
2. Contact individually each potential evaluator to introduce the project and describe their role.
3. Send an email containing the intervention protocol and recordings to interested evaluators. • Ask the evaluator to get acquainted with the material before taking the next step.
4. Have evaluators complete the first questionnaire on Lime Survey online (link in an email with intervention protocol). • Have them sign the consent form. • Have them answer questions.
5. Summarize the answers from the first round. • Conduct quantitative descriptive analysis (using Excel file and tables). • Validate the categories of responses obtained with the QDA miner software. • Write the second questionnaire based on the responses obtained (suggestions and non-consensual items).
6. Have the evaluators complete the second questionnaire using Lime Survey.
7. Synthesize the quantitative and qualitative data.

#### Measures

We first designed a customized questionnaire with a total of 27 questions: 5 questions about socio-demographic information (gender, age range, region, occupation), 8 questions on feasibility and preliminary acceptability of the interventions, 14 questions assessing the three interventions and their components. Each question was rated on an agreement likert scale (strongly agree, somewhat agree, somewhat disagree, strongly disagree, and “don't know”). One question was rated on a yes/no scale. We invited evaluators to provide comments for each question (see Table 4).

**Table 4:** Questionnaire 1.

	**Strongly agree**	**Somewhat agree**	**Somewhat disagree**	**Strongly disagree**	**I don't know**		**Blank**
**Preliminary program feasibility and acceptability**							
1. Managing pain, anxiety, and increasing	18 (82%)	4 (18%)					
wellbeing in palliative home care patients are issues that warrant non-pharmacological intervention.	100%						
2. The intervention will have the desired effect on	8 (36%)	13 (59%)			1 (5%)		
pain, anxiety, and wellbeing of patients.	95%						
3. In your opinion, patients will be interested in	6 (27%)	15 (68%)	1 (5%)				
participating in the intervention.	95%						
4. The intervention program will fit well into the	11 (50%)	11 (50%)					
daily life of the consumers and will be easy to use.	100%						
5. With a short training session, the field workers	14 (64%)	7 (32%)	1 (5%)				
will have the necessary skills to enable the implementation of the intervention.	95%						
6. There will be good cooperation from staff and	9 (41%)	10 (45%)	1 (5%)		2 (9%)		
family caregivers in implementing the intervention. If no, please indicate the elements that will ensure the cooperation of the staff and caregivers.	86%						
7.What are the facilitators and barriers to implementing this program? Explain.	Comments only				
	No		Yes				
8.The intervention program may result in negative effects on patients.	15 (68%)		7 (32%)				
	**Strongly agree**	**Somewhat agree**	**Somewhat disagree**	**Strongly disagree**	**I don't know**	**I did not evaluate this intervention**	**Blank**
**General evaluation of interventions**							
1. The hypnosis intervention is well-constructed and	9 (50%)	8 (44%)	1 (6%)			3	1
forms a coherent whole.	94%						
2. The music intervention is appropriate and	7 (58%)	4 (33%)	1 (8%)			9	1
coherent for the patients.	92%						
3. The hypnosis and music intervention as a	6 (46%)	7 (54%)				8	1
whole is coherent and appropriate for the patients.	100%						
4. Voice rate, timbre, pauses, and other sound	12 (57%)	7 (33%)	1 (5%)		1 (5%)		1
parameters are appropriate.	90%						
**Assessment of intervention components**							
1. The intervention's set-up by the on-site provider	11 (58%)	7 (37%)			1 (5%)	3	
and its introduction are adequate and will promote the patient's adherence to the intervention.	95%						
2. The “introduction,” “induction,” “safe place” and	6 (32%)	10 (53%)	1 (5%)		2 (11%)	3	
“deepening” sections are appropriate (vocabulary, content, etc.) and will be appreciated by palliative care patients aged 65 and over.	84%						
3. Metaphor 1 of the horse is adapted to patients	10 (59%)	6 (35%)		1 (6%)		3	2
(vocabulary, content).	94%						
4. Metaphor of Island is appropriate for consumers	7 (47%)	6 (40%)		1 (7%)	1 (7%)	4	3
(vocabulary, content, etc.).	87%						
5. Metaphor of “reflection” section of the	8 (57%)	6 (43%)				5	3
second-guided imagery session is user-friendly (vocabulary, content).	100%						
6. Metaphor of the positive hand technique is	6 (46%)	5 (38%)	1 (8%)		1 (8%)	6	3
user-friendly.	84%						
7. The sections “post-hypnotic suggestion” and	8 (42%)	4 (21%)	3 (16%)		4 (21%)		3
“emergence” are appropriate and adapted to the patients.	63%						
**Music intervention components**							
1. Background music detracts from the effect of		2 (14%)	4 (29%)	5 (36%)	3 (21%)	5	3
interventions with music			65%				
2. Inserting preferred music detracts from the effect			5 (33%)	9 (60%)	1 (7%)	4	3
of the intervention			93%				
3. Text in the intervention “music” (induction,		1 (7%)	3 (21%)	8 (57%)	2 (14%)		3
deepening, emergence, etc.) decreases the effect of the preferred music.			88%				

*The color blue corresponds to responses in favor of the initial intervention, taking into account the positive or negative wording of the question*.

Based on the results of this first questionnaire, we developed a second questionnaire with 40 questions. Fifteen questions were addressed to all evaluators: six questions focused on the potential negative effects of interventions, two on the interpretation of the text, three on the language used, and four on the modalities of the intervention. Twenty-five questions focused on specific content and were primarily intended for hypnotherapists, although other reviewers could provide input if they wished. Evaluators could add their comments after each section.

#### Analyses

Questionnaire responses were analyzed quantitatively to assess program acceptability and feasibility. Unanswered and non-applicable (“I did not evaluate this intervention”) questions were not counted to calculate response rates. In the literature, there is currently no consensus as to what percentage to use. Some authors interpret a percentage of 80–100% as a strong consensus (35). Based on this range of recommendations, the level of consensus for the first questionnaire was set at 80% agreement (strongly/somewhat agree or strongly/somewhat disagree). As a conservative measure, we considered in the calculations the answers “I don't know,” as “strongly/somewhat disagree.”

The comments collected from the evaluators were then compiled to extract their opinions. We performed an initial global reading of all answers in the first questionnaire to capture the full diversity of themes covered in the comments. We then divided the content into categories. To ensure that all comments were grouped according to the established themes, a content analysis was performed on these data using the QDA miner lite software (36). Two authors (SP and ATJD) validated the categories and content associated with each category. For the last step, we reread the content of each category several times to extract the overall meaning, before summarizing it. We also illustrated the content of the category by selecting one or a few comments representative of the opinions expressed.

For the second round, we first examined whether the non-consensual items in the first questionnaire received a sufficient level of agreement. We then analyzed the comments following the same procedure as for the first questionnaire.

## Results

Quantitative data with their qualitative content are presented in the following section for each of the questions asked. Results related to the preliminary feasibility and acceptability are presented first, followed by results related to the content of the interventions.

### Preliminary Feasibility and Acceptability of the Intervention Program

The evaluators consensually rated the preliminary feasibility and acceptability of the program as adequate. Agreement was over 85% for all items except one concerning the risk that the intervention might cause negative effects among palliative care patients. Particular attention was paid to this aspect.

#### Relevance of the Program

According to experts, managing pain and anxiety and increasing the wellbeing of people in palliative care justifies the presence of a non-pharmacological intervention program. Indeed, several evaluators commented (*n* = 6) that pharmacological interventions alone are not effective in controlling pain and anxiety in people with palliative care. All physicians commented that polypharmacy issues warranted additional interventions.

#### Expected Effects of the Program

Almost all evaluators (95%) believed that the program would produce positive effects on pain, anxiety, and wellbeing. The interventions can bring help “*to relieve the body, to relax morally, to take a break to regain strength or to better control certain pains”. (C2)* (Alphanumeric codes after the citations refer to the profession-competency of the evaluator, as described in Table 2).

#### Potential Interest of Patients

The evaluators (95%) believe that palliative care patients will be interested in taking part in the intervention. We received several suggestions for increasing patient interest and engagement: taking the time to clearly explain the project and demystify the intervention, clarifying the patients' expectations and reassuring them about their fears, involving the professionals who care for the patient in the process, and helping them with the process and application. Evaluators also reported that providing examples of positive experiences to patients could positively impact their interest in the program. Besides, they mentioned that adherence to the intervention may depend on the way the practitioner and family members present it, and their motivation to improve the care and quality of life of people in palliative care.

*(…) I believe that if the staff adheres to the project, they become facilitators, since they are the ones who will “sell” the project to the patient. If the staff is convinced of the merits of the project, the patient will quickly be convinced. In a vulnerable situation, we quickly defer to the opinion of the person who is helping us. (N3)*

The attitude of staff and caregivers can actually help or hinder interest in the program.

*In my opinion, the accompanying person must be empathetic, calm, and patient, in order to comfortably accompany people on their journey. (N2)*

However, the patients' condition is a factor that may work in favor or against the realization of the program. In the context of end-of-life, it was mentioned that some persons may change their minds due to a multitude of factors beyond their control. Attention and concentration skills may also be reduced and some patients with severe physical and cognitive impairments may not be able to participate in the interventions. An emergency or intense uncontrolled physical pain could prevent the person from being physically and mentally available for the content of the experience. These imponderables are part of the reality of people at the end of life.

#### Ease of Implementation of the Program

All evaluators agreed that the intervention program will be well-integrated into the daily lives of patients and that it will be easy to use. They emphasized the facility of implementing the intervention its flexibility, and the fact it could potentially be used independently by patients with no severe physical or cognitive impairments.

The technology aspects were also discussed. One evaluator mentioned that some patients may have hesitations to using technology to listen to interventions. Being forced to use headphones could also interfere with the intervention. As such, it was suggested that a small speaker could be used if necessary. Other evaluators (27%) gave positive comments about the ease and the flexibility in the use of the material. One mentioned that “*the pre-recorded treatment can be easily adapted to the beneficiary's schedule and will facilitate its implementation and the adherence of the palliative care patients” (N2)*. Others mentioned “*It requires little equipment and preparation time* ++” *(BA)*, and “*I can see it fitting in very well when patients feel they need a rest and quiet time” (N1)*.

Some evaluators (22%) noted that the intervention length was considered too long, while others considered it was adequate. In response to our second questionnaire, experts indicated that the ideal length of the intervention would be 20 min (61% of respondents) and 25 min (72%).

#### Training

Most evaluators (95%) considered the short training offered to health care providers (nurses, social workers and beneficiary attendants) to be sufficient to facilitate the implementation of the intervention “*The process is quite simple, so it is easy to implement for the health care providers” (SW2)*.

Conversely, other evaluators had a different opinion. On the one hand, one evaluator (4%) indicated that this training was not necessary for program implementation. On the other hand, two of them (9%) emphasized the need for more training to be able to respond to patients' hesitations and to develop interpersonal intervention and listening skills to support the potential emotions generated by the music and imagery.

*How can we ensure that the workers on the site will have the training to accompany the person who expresses the emotions evoked by the music or imagery? (MT1)*

#### Cooperation of Staff and Caregivers in Implementing the Intervention

Most evaluators (89%) felt that the cooperation of staff and caregivers would be good. Adherence to the intervention, the quality of the caregivers' training (clear explanations, relevance), the support offered, and the time required of the caregivers are aspects that were raised as potentially influencing the cooperation of the staff.

*The cooperation of the personnel always depends slightly on the time it takes and the adherence of a person in this type of intervention. Time can sometimes work against the professionals. For family caregivers, I believe that it will be easy to support the implementation of the intervention, except for the type of personality that is hesitant or does not believe in the approach. (N2)*

According to another evaluator, the most significant barrier may be overworked staff and their flexibility to change an already established routine. On the administrative side, one evaluator mentioned that strong commitment is important to facilitate the program implementation. They also note that limited financial issues could be a hurdle to program integration by delaying or preventing its completion.

The simplicity of program implementation may act as a facilitator for the program. Indeed, it was considered “*very clear, well described and easy to apply and pleasant to apply with a patient*” *(P2)*. This implies possible benefits for health care providers (e.g., satisfaction) that may reinforce their engagement in the program.

#### Potential Negative Effects

Evaluators were asked if the intervention program could have negative effects on patients. One third of evaluators (32%) answered in the affirmative. Potential risks mentioned included:

- the pain could be increased when a feeling of heaviness is evoked in the induction part;
- the patient could have difficulty choosing a pleasant place, leading to a feeling of incompetence, a sense of failure or unhappiness;
- the music may evoke strong emotions;
- the images perceived could be different from those suggested which could create distress.

To reduce the risk of negative effects, we addressed these points in the second questionnaire.

Most of the evaluators (65%) agreed, in this second questionnaire, that adding alternative sensations, in addition to “heaviness” (e.g., the body could feel like it was floating), could decrease the risk that there would be an increase in pain associated with the word “heaviness.” They also agreed (91%) that asking the patient to choose a pleasant place before the session and adding statements normalizing experiences in which they might not have visual, auditory, or other sensations might lower the risk of the patient experiencing a sense of failure.

To reduce the risk of unmanageable emotions related to listening to music, evaluators recommended that certain precautions be taken when selecting pieces. They suggested asking the patients to consciously choose a piece that generates a positive, pleasant, and wellbeing emotion in them and to make sure that this music does not refer to traumatic memories or too high negative emotional charges. Evaluators also proposed to verifying the musical content (lyrics, harmonic and melodic structure, mode, tempo, emotional content) of the chosen pieces. In the second questionnaire, the majority of evaluators (75%) indicated that these measures would significantly reduce the risk of uncontrollable negative emotions related to the chosen piece occurring during the intervention. Several evaluators (40%), however, expressed doubt that the intense emotions that could be generated by the music could be a negative element for the palliative care patients, as illustrated in the following remarks.

*I agree that letting the client choose the music can only be helpful. However, I believe that grieving is also experiencing negative emotions at times, and experiencing this anger and sadness is actually helpful for the person. I don't think we should try to protect them from their own emotions, quite the contrary. We must be careful that our own fear of suffering does not taint this intervention. (N-H)*

*I also believe that the so-called'negative' emotions that may surface are sometimes a necessary part of the process and can help release and reduce anxiety. These “negative” emotions are often more disturbing for those around them than for the person. (BA)*

Finally, one evaluator mentioned the possibility that images perceived by patients may be different from those suggested and that a patient may experience images of their death that could cause distress. This question was addressed and commented on by the evaluators in the second questionnaire:

*(…) if a person is imagining their own death and it's causing them strong emotions, I think it's a good thing to release that. As long as the helper is comfortable with the emotions and just being present. (HT5)*

Despite the different opinions about the risks that music or images can generate strong emotion and distress, 70% of the evaluators agreed that a procedure for managing emotions would significantly decrease the likelihood that significant distress would persist beyond the session. One evaluator suggested that follow-up with an outside provider be offered, if necessary.

In summary, several suggestions were made and evaluated to decrease the risk of negative effects. When applying all the above measures, 90% of the evaluators disagreed that the intervention could have negative effects.

#### Other Considerations

Two evaluators (caregivers) noted that they fell asleep while listening to the intervention. In the second questionnaire, we asked what the best strategy would be to manage this type of situation. Ninety percent of the evaluators agreed that the time of naps should be checked so that the intervention would not take place at that time.

### Evaluation of the Interventions

Program and content evaluations of each of the three interventions (H, M, and HM) showed that they were appropriately conceived and coherent. However, evaluators proposed several points of improvement for each of the components.

#### Setting Up the Intervention

In the first part of the protocol, we proposed a procedure for the intervener who will be on the site during the intervention sessions (i.e., at the home of the patient). It includes three steps: introduction to the intervention, preparation of the patient, and listening to the intervention. At the end of the intervention, the intervener concludes the session.

This protocol for the implementation of the intervention was evaluated as adequate and favorable to the patient's adherence to the intervention. It was considered “*simple and easy to carry out” (C1)* and the steps “*are well explained and clear” (FC1)*.

#### Hypnosis Intervention

Evaluators positively rated the hypnosis intervention. A nurse hypnotherapist noted, “*Very nice script, very well structured and clear path. Lots of interesting material to experiment with it*.” Another evaluator noted it was “*easy and pleasant to listen to and led to greater relaxation. It is easy to follow the voice and see yourself in peaceful places where there is great well-being.” (N1)*

A third nurse, however, indicated that she “*found it difficult not to have music in the hypnosis sessions, it brought me less into a deep relaxation” (N2)*.

Suggestions related to language were offered. A speech therapist evaluator indicated that hypnosis interventions could be cognitively demanding for people in palliative care because of the number of verbal utterances. This person suggested limiting the number of utterances and encouraging simple sentences and silences. We addressed this point in the second round, and 92% of the evaluators agreed with his suggestion.

At last, more than 80% of evaluators rated all but one section of the script as appropriate for palliative care patients. The emergence section received <80% agreement. This was carefully considered. Comments to improve the content of interventions were given as detailed below.

##### Introduction

The introduction proposed that “*curiosity and openness allow you to discover new things, to be fascinated by what is going on, to keep a certain sparkle about life.”* An evaluator suggested avoiding the term “sparkle about life,” as well as the term “deep joy,” “*which might bring a mixed or complex feeling” (P3)*. Evaluators (73%) agreed with removing this phrase.

##### Induction and Deepening

In the induction phase, it was indicated to look at a fixed point without closing the eyes. Four evaluators (two family caregivers, a social worker and a nurse) indicated that this sentence generated a feeling of frustration in them. Not being able to keep their eyes open made them doubt their ability to fully experience the intervention. “*You wonder, for several minutes, if the experiment will be valid, if you miss this condition” (FC1)*. It was therefore suggested that patients should not be asked to keep their eyes open. This suggestion was accepted by 75% of the evaluators in the second round.

In the induction, which contains elements of relaxation and release, a hypnotherapist indicated that it was important “*that the person can give himself/herself the right to be in pain.”* It was suggested that “*there is dissociation between two parts of the self, one that has pain and one that does not, and that the first part be allowed to have pain in its own way while another can relax*.” The evaluators (70%) agreed with this sentence.

The original deepening included a 0–10 count. One evaluator indicated that it had too many words and that it was better to use a count to five only. This proposal was accepted by 91% of the evaluators.

##### Pleasant Place

In the intervention, patients are asked to imagine a pleasant place. The script took care to bring this pleasant place to live by directing attention experiences therapist to the different sensory modalities. It was mentioned that it would be appropriate to introduce different sensory modalities earlier on, to ensure that each individual feels concerned quickly, regardless of which sensory mode they prefer.

*Using different sensory modes is fine. However, it's important to introduce the different modes at the beginning so that you don't lose too many people along the way (…) Doing smaller loops for each sensory mode could be helpful (instead of long loops). (HT4)*

Evaluators, including four hypnotherapists, agreed (64%) with this proposal.

##### Horse Metaphor

Nearly all evaluators (92%) indicated that the horse metaphor was appropriate for patients (vocabulary, content). In addition, evaluators indicated that the metaphor allowed for letting go and was beautiful.

*It allows the patient to move towards letting go, to understand that sometimes letting go of the reins and letting go of your body, your intuition, letting go of life allows you to get to the right path…to let go of resistance and move towards trust. (N2)*

However, it was noted by a speech therapist and family caregiver, that the metaphor was a bit long and that it could be demanding for a person with cognitive limitations.

##### Island Metaphor

This metaphor was deemed appropriate for patients (vocabulary, content) by 87% of evaluators. However, the aim of the metaphor was rated as more or less clear.

*I understand this metaphor less well. Either one chooses the quicker, but the more painful path (and at the same time, the person would be proud of himself), or one takes his time, and it is gentler to get to the Island. (HT5)*

##### Duration and Density of Interventions

In the first questionnaire, four evaluators, two physicians, and two hypnotherapists, indicated that the duration and density of the hypnosis intervention might be too high.

*For the first session, it would be good not to put too many elements. Currently, the session is very busy, too dense. I suggest removing the island metaphor or removing the island metaphor and the horse metaphor. Maybe a little long, not much room for silence and pauses. (HT2)*

To reduce the length of a session, 44% agreed with removing the horse metaphor, and 63% agreed with removing the island metaphor.

##### Reflection

All evaluators indicated that the “reflection” section of the second-guided imagery session is appropriate (vocabulary, content). It was felt that this metaphor allowed the subjects to make sense of what they were experiencing and to see them taming their experience allowed them to “*regain confidence in their ability to be well and to regain the memory of a state of comfort and well-being (…) to appreciate themselves” (HT2)*, “*to make sense of what the subject is experiencing” (HT5), “to appropriate peace and* serenity for *themselves” (SW2)*, and “*to find them interesting and positive” (FC1)*.

It was suggested that some kinesthetic elements be added to the “*reflection*” section so that it could be experienced more deeply.

##### Positive Hand

The evaluators (84%) rated the positive hand technique as adequate for patients. They indicated that the technique helped calm anxiety, feeling comfort and relief, and taking care of oneself. However, one evaluator noted that “*this technique is a bit longer and the description seems a bit more abstract, possibly more difficult to access for some patients in more concrete thinking (…)” (P1)*.

To improve the technique, evaluators suggested giving more guidance when the light hand is placed on the part of the body that needs it, to better accompany this effect. It was proposed to connect the two parts, to create even more lightness so that the hand would start to float and then be strongly drawn to the part of the body that needs comfort. These suggestions were accepted by 78% of the evaluators (including 3 hypnotherapists).

##### Post-hypnotic Suggestions

Most of the evaluators (63%) felt that the post-hypnotic suggestions and emergence section were relevant. This percentage is lower than the 80% established for the first round. Therefore, we looked at this point conscientiously in the second round.

One of the post-hypnotic suggestions was to use a dimmer switch to manage pain levels. One evaluator suggested letting the person choose the part of the body where they wanted to put their switch and 70% agreed with this suggestion.

In the text, it was also written: “*The heart returns to a beat of normalcy*.” This sentence alluding to normality was considered tricky by a family caregiver. The evaluators (91%) agreed that it would be better not use terms referring to normality.

In addition, two evaluators objected to the use of the word emergence in the subheadings of the scenario, thinking that patients would read it. This partly explains the low agreement rate in this section.

#### Music Intervention

Most evaluators (92%) felt that the music intervention was appropriate and consistent. They noted that the music intervention was “*easy to follow” (P2) “focused, relaxing, and easy to perform” (P1). “It allows for even more well-being” (SW2)* and is an “*important contribution and helps to relax” (N1)*. It “*evokes positive emotions, gives energy, adds ideas” (FC4). “I love what the music brings to the experience!” (HT5)*.

One evaluator (N-H), however, raised the possibility of a break between the rhythm of the spoken text and the music. The calm tone and script that leads to deep relaxation juxtapose with the “*jovial, inspiring*” music of pieces that may be chosen.

In the comments, it was suggested to remove the emergent section after the music. “*We don't need feedback after listening to a piece. Therefore, I propose that the intervention ends with the preferred music, without feedback from the voice” (HT4)*; Thirty-nine percent agreed with the proposal and 39% disagreed.

##### Contribution of Selected Music

More specifically, 93% of the evaluators judged positively the integration of music chosen by the patient him/herself. It allows the patient to be a stakeholder in his or her intervention, which “*will be a way of ensuring the patient's collaboration in this intervention” (FC1)*.

Some evaluators also mentioned that hearing a song they knew was reassuring, that it “*brought back good memories” (BA)*.

*I'm all for favorite music, making the overall intervention more acceptable and enjoyable. I think it would help the patient do the procedure again more often. (P1)*

If the person does not have music in mind, it was suggested to “*add music choices that take them into a zone of memories and tenderness (e.g., Brahms's Lullaby, Goodnight) (…) that many grandmothers sang fondly to their children and grandchildren or other tunes from that time” (N1)*.

##### Contribution of Background Music

In both the Music and the Hypnosis/Music interventions, background music was included during the phase of induction, deepening, post-hypnotic suggestions and emergence phases. We asked evaluators to comment on the following sentence: “*Background music detracts from the effect of interventions with music*.” Most of them (65%) disagreed with this sentence. This percentage is smaller than the targeted 80% agreement. Nevertheless, 10 evaluators gave comments, and these comments were positive about the musical background. The background music was rated as “*pleasant, relaxing and soothing” (P2), “It helps to wrap the person in softness” (FC4), “for me the music adds to the effect, the music carries me!” (HT5), “I like the background music. I wouldn't take it off” (P1)*.

#### Hypnosis and Music Intervention

All the evaluators agreed with the following statement: “*The imagery and music intervention as a whole is coherent and appropriate for the patients*.” Background music and hypnosis “*complement each other well I think” (SW2). “I really like the combination of the two” (N-H)*, it “*continues the effect of hypnosis. “The imagery and the music form an ensemble of great peace and well-being, that's how I experienced it” (N1)*.

On the other hand, 88% of them disagreed with the statement “The text in the music intervention (induction, deepening, emergence, etc.) diminishes the effect of the preferred music,” as indicated by this comment:

*I find that the text allows us to better appreciate the music afterward (P1)*.

Finally, given that the hypnosis and music intervention integrate components of the hypnosis intervention and of the music intervention, we consider that the suggestions made for each intervention separately may also apply to the combined intervention.

#### Interpretation of the Text

The way the text is delivered can influence how it is received. For this reason, we asked some questions related to the interpretation of the text, to make the necessary modifications. Most evaluators (90%) considered adequate the voice rate, timbre, pauses and other sound parameters. Nevertheless, some of them suggested more pauses and slower speech. This opinion was shared by 68% of them regarding the number of pauses and by 42% for the flow of the voice.

## Discussion

Program development research emphasizes the importance of the early stages of development prior to evaluation studies (37). As such, we defined the design of the MuzHyp^©^ program and conducted a mixed-method study to refine the Program according to ORBIT-Phase Ia and Ib, respectively (26).

To meet those objectives, we conducted a consultation process that demonstrates the relevance, feasibility, and acceptability of the program and highlights the evaluators' recommendations for improvements.

### Design

In accordance with ORBIT-phase 1a, we searched the literature for evidence-based procedures using hypnosis and/or music in the management of pain, anxiety, and wellbeing in palliative care patients. This design definition is an essential phase in the development of new programs, as behavioral science recommends improving existing procedures rather than creating new ones (37). Now that this protocol has been defined based on the identified studies, it was also important to define a program appropriate for a Quebec palliative care patient population. For this reason, in accordance with the ORBIT phase 1b model, we conducted a redesign study (26).

The evaluators consider that the intervention program meets a need in the palliative care population. They believe the implementation of the intervention to be simple, accessible, and requiring limited material, human and time resources. They assessed the content of each intervention as adequate, consistent, and appropriate for palliative care patients.

The expected effects of the interventions are positive and the need for non-pharmacological methods to manage pain, anxiety, and wellbeing was emphasized by the evaluators. These data are consistent with the issues raised about medication in palliative care (4).

According to the evaluators, participant interest is a key factor that may play a role in the effectiveness of interventions. In this regard, they raised several avenues to enhance their interest in participating in the intervention. This interest can also be promoted by the possibility of adapting the program according to the choice of intervention the patient wishes to experience (hypnosis, music, or music with hypnosis), their musical preferences, and the pleasant place they wish to explore. This flexibility respects the principles of person-centered medicine interventions (29). Besides, the ease of implementing a program was found to be a factor that could play a role in the acceptability of the intervention. The evaluators considered both the technology and the intervention program to be simple and flexible. The positive consensus reached on these issues by diverse actors, including caregivers, health care professionals and health service managers coordinating at-home palliative care setting is encouraging.

### Redesign

In program development studies, it is essential to validate the design with patients or health professionals (38). In this project, we refined the intervention program considering the quantitative results of the consultation process as well as the comments of 22 evaluators from different professional fields with distinct socio-demographic characteristics.

#### Preliminary Feasibility and Acceptability

##### Training Manual and Cooperation of Health Care Providers

The evaluators identified several elements to support the cooperation of health care providers and family caregivers. The intervention should fit easily into their routine. The quality of training and the time required are also among the factors that can influence cooperation and the preliminary feasibility and acceptability of the intervention. Besides, caregivers are often faced with limited time resources and high levels of professional stress (39). It is therefore important not to overburden them with complex and time-consuming training and interventions.

Based on this feedback, we wrote an intervention manual, paying particular attention to the length of the training, the simplicity and the clarity of the intervention program description and the procedures to be followed. Considering the evaluators' recommendations, we also included the expected benefits and some positive testimonials, as well as recommendations to counter any hesitation and address patient concerns. Finally, in the intervention manual, we have added some basic recommendations on how to interact with palliative care patients in this program.

##### Duration/Density of the Sessions

The length of each session and its density were identified factors that can affect the feasibility of the program. The evaluators were sensitive to the importance of not tiring patients. For this reason, the maximum duration of the intervention was set at 25 min and the content of the interventions was reduced.

To reduce the density of the sessions, we removed the island metaphor. We also trimmed the text by reducing the number of words and favoring simple sentences.

##### Risk Reduction

The evaluators identified some potential risks of the interventions, including the risk of experiencing negative emotions. However, there was no consensus on this topic. Some of them considered that negative emotions, and images of one's own death are a normal process at this stage of life and that it would even be beneficial to experience them. Therefore, measures to minimize them would not necessarily be the most helpful for these individuals. Other evaluators, however, consider that too much negative emotion can lead to unwarranted distress. Considering that no negative effects have been found in studies of music or hypnosis in palliative care (25), we put some measures in place, while trusting the choices of palliative care people regarding the pleasant place and the musical pieces they wish to experience. As a preventive measure, during a preliminary meeting, we will ask the patients to identify a pleasant place and choose musical pieces that generate in them pleasant, positive emotions in addition to bringing them wellbeing. We will then verify that the pieces are not associated with painful or negative events, by asking them what it reminds them of and by checking if these pieces can generate a negative emotion in them. After the meeting, the first author will verify their content. If certain risks are identified, we will warn the interveners who will be on-site to be attentive to the participant's reactions, and plan additional meetings with qualified staff, to help him/her, if necessary. In order to work on risk reduction upstream, we have integrated some listening techniques to the care worker's manual that will be explained during the training.

Among the potentially negative effects identified, the evaluators noted the possibility of increasing the sensation of pain through the use of the word “heaviness.” To reduce this risk, we added to the “heaviness” sensation, alternative sensations to be felt (e.g., lightness, looseness). It has been evaluated that this way of doing would allow the patient to choose the sensation he prefers and thus reduce the risk of increasing the pain.

To prevent participants from falling asleep, we take care not to give the intervention during their usual nap time. To encourage waking up if drowsy, we added three bell sounds at the end of the intervention. In case of falling asleep, it was decided to gently wake up the person 10 min after the end of the intervention.

#### Content

Following the various suggestions, we have removed the sentences referring to sparkles about life and normality. We also removed the sentence mentioning that patients should not close their eyes while staring at the dot. This was done to avoid showing doubt about their ability to experience the sessions properly.

Considering the probability that pain may persist despite the induction of a state of relaxation, we have added a sentence indicating that it is possible to feel less pleasant sensations while other areas of the body relax at their own pace. Then, to deepen the state of relaxation and letting go, we counted down to 5, instead of 10.

In response to the various suggestions related to the transformation section, we addressed the different sensory modes more quickly at the beginning of the “pleasant place” section. We slightly reduced the length of the horse metaphor, added kinesthetic elements to the reflection metaphor, and added guidelines to the positive hand metaphor to better accompany the hand as it lands on the body part that needs it. Finally, for the switch metaphor, we leave it up to the patient to place it where they wish.

In the comments, it was suggested that the emergence section be removed after the presentation of the chosen music. Since opinions were divergent on this point, we finally adopted the recommendations of music therapists who advocate keeping the emergence after the musical piece.

Finally, the contribution of background music reached a mixed level of consensus. Considering that all comments were contrary to the quantitative results, we suspect that the negative wording of the question might have misled some respondents. It is also possible to explain this result by the potential preference of some people to listen to the text alone, without musical background.

### Internal and External Validity

We believe that the careful process of developing the intervention according to the ORBIT recommendations, the development of a manual to be used in the next phases of experimentation, and the presence of pre-recorded sessions will promote a good level of internal validity and reliability. The development of the study in collaboration with the host community, ensuring that the intervention is feasible and applicable “in the real world” will, in turn, promote the achievement of a certain level of external validity for future studies (ORBIT-Phase II). We think that the balance between external and internal validity in the development of the intervention program is an advantage both for future clinical implementation and for research.

### Expected Outcome

This intervention program took care to consider some important issues related to the pain, anxiety, and wellbeing of patients in palliative care. It was inspired by those of Gutgsell et al. (21), Peng et al. (23), and Coelho et al. (40), which revealed positive effect sizes on pain, anxiety, and wellbeing. Given that the baseline interventions have been shown to be effective, we believe that MuzHyp^©^ Program refined in this study also has the potential to achieve the desired effects on the palliative care population.

### Limits and Strengths

We noted some limitations to consider in our study. First, a limitation could be the representativeness of the evaluators recruited. They are mostly 35–49 years old, female, and all come from the province of Quebec.

Second, the pre-recorded format of the intervention program can be both a limitation and a strength of the program. Unlike interventions where a therapist is present and can adapt in real time to the patient, the pre-recorded intervention is less flexible in terms of its content and possible variations. We may expect that face-to-face intervention with a therapist could potentially yield more important results and that in clinical terms, in-person interventions are recommended. Nevertheless, in a palliative care context where resources are limited and where individuals may have needs at different times of the day, having an accessible, easy-to-use tool can also contribute to its implementation, and increase member buy-in while complementing other services offered.

Finally, the development of a program, according to validated and well-defined standards as the ORBIT model, makes it possible to offer more targeted interventions. It also makes it possible to respect the time and material resources of both the receiving environments and the palliative care patients.

## Conclusion

In conclusion, this program was assessed as feasible and acceptable, and its content was found to be adequate. The proposed amendments have contributed to increased feasibility and preliminary acceptability, including reducing the perception of potential adverse effects. We are confident that this standardized process has helped to improve the quality of the content offered. The next step will be to evaluate the feasibility, acceptability, and effectiveness of this program with patients in palliative care at home, in a pilot efficacy study. This research provides essential milestones for the successful development and integration of music and hypnosis as complementary approaches to personalized comfort care delivered at home.

## Data Availability Statement

The raw data supporting the conclusions of this article will be made available by the authors, without undue reservation. Those interested in the details of the intervention protocol may contact the first author directly.

## Ethics Statement

The studies involving human participants were reviewed and approved by Ethics Committee of University of Montreal -CERSE (# 2021-1243) and CISSS-Chaudières-Appalaches (# 2022-896). The patients/participants provided their written informed consent to participate in this study.

## Author Contributions

JB, PR, and DO designed the protocol and secured funding to conduct the study. JB, SP, AD, and DO were involved in the design of the questionnaires. SP and AD participated in the validation of the categories and their content and A-MP, DO, and JB participated in the recruitment of evaluators. JB conducted the assessment process and the quantitative and qualitative analyses. JB wrote the report with PR and DO and all authors commented on or approved the final version of the manuscript.

## Funding

This work was supported by Mitacs no. IT25481 in partnership with Recherche-Interventions Cétosia.

## Conflict of Interest

This article was completed as part of a Mitacs funded postdoctoral fellowship in partnership with Recherche-Interventions Cétosia, a company founded by JB. Conflict of interest controls are in place at the business incubator that supervises JB. The remaining authors declare that the research was conducted in the absence of any commercial or financial relationships that could be construed as a potential conflict of interest. The handling editor NK-M declared a shared affiliation with the author AD at the time of review.

## Publisher's Note

All claims expressed in this article are solely those of the authors and do not necessarily represent those of their affiliated organizations, or those of the publisher, the editors and the reviewers. Any product that may be evaluated in this article, or claim that may be made by its manufacturer, is not guaranteed or endorsed by the publisher.

## Footnotes

^1^Ogez D, Aubin M. *Pratiquer l'autohypnose dans la gestion de la douleur chronique. Hypnose et thérapies brèves*.
